# Prevalence and Types of Anemia in Patients With Hypothyroidism in Buraidah, Saudi Arabia

**DOI:** 10.7759/cureus.112269

**Published:** 2026-07-08

**Authors:** Abdulrahman Alharbi, Omar K Alsaawi, Saleh A Alhablen, Marwan K Aljumah, Ibrahim K Alharbi, Moath K Alshowaish

**Affiliations:** 1 Department of Diabetes and Endocrinology, King Fahad Specialist Hospital, Buraidah, SAU; 2 Department of Internal Medicine, King Fahad Specialist Hospital, Buraidah, SAU

**Keywords:** anemia, chronic disease, hematologic indices, hypothyroidism, microcytic anemia, normocytic anemia, thyroid dysfunction

## Abstract

Background

Anemia is a common and often underrecognized comorbidity in patients with hypothyroidism. Its etiology is multifactorial and is influenced by thyroid hormone deficiency, nutritional status, and concomitant chronic conditions. Understanding the prevalence, hematological patterns, and predictors of anemia in patients with hypothyroidism is necessary to improve clinical outcomes. This study aimed to assess the prevalence of anemia, describe its types and features, examine its association with thyroid hormone status, and identify the independent predictors of anemia in patients with hypothyroidism.

Materials and methods

A retrospective study was conducted in 444 patients diagnosed with hypothyroidism. Demographic, clinical, and laboratory data were extracted from the electronic medical records. Hematological indices were compared between the anemic and non‑anemic groups using independent t-tests and chi-square tests. Multivariate logistic regression was performed to identify the independent predictors of anemia after adjusting for age, sex, diabetes, and hypertension.

Results

Anemia was present in 112 (25.2%) patients. In anemic patients, the most common type was normocytic anemia, occurring in 64 (57.1%), followed by microcytic anemia in 46 (41.1%) and macrocytic anemia in two (1.8%). Patients with anemia had significantly higher platelet counts than those without anemia (p < 0.001). Markers of thyroid function (TSH, T3, and T4) did not differ significantly between the groups (p > 0.05). Multivariate analysis revealed that female sex (adjusted odds ratio (AOR) = 3.210, 95% confidence interval (CI): 1.323-7.789, p = 0.010) and the presence of chronic diseases (AOR = 3.458, 95% CI: 1.829-6.536, p < 0.001) were independent predictors of anemia.

Conclusions

One in four patients with hypothyroidism had anemia, and the most common type was normocytic anemia. No significant differences in thyroid hormone levels were observed between anemic and non-anemic patients. Female sex and chronic diseases were significant risk factors for anemia, emphasizing the importance of targeted screening and comprehensive evaluation of these subgroups. Early diagnosis and treatment of anemia may improve overall clinical outcomes in patients with hypothyroidism.

## Introduction

Hypothyroidism is a common endocrine disorder caused by insufficient production of thyroid hormones, resulting in systemic metabolic and physiological derangements. It occurs in 4-10% of the population worldwide, with a higher prevalence in women and the elderly [1]. Hypothyroidism is increasingly recognized as a public health problem in the Arab regions, including Saudi Arabia, with prevalence estimates varying from 11-15% based on population characteristics and diagnostic criteria [2].

Thyroid hormones are essential for erythropoiesis and regulate erythropoietin production, iron metabolism, and bone marrow activity [3]. Thus, thyroid hormone deficiency may adversely affect RBC production and maturation, thereby predisposing patients to anemia. Several mechanisms can explain this association. Macrocytosis may be caused by vitamin B12 deficiency and pernicious anemia in patients with autoimmune thyroid disease [4]. Normocytic anemia may be caused by decreased erythropoietin secretion and bone marrow suppression [5]. Microcytic anemia may be caused by impaired iron absorption or chronic blood loss [6]. Unrecognized hematologic abnormalities may complicate clinical assessment and delay appropriate management because the symptoms of anemia overlap with those of hypothyroidism.

Recent international studies have highlighted the clinical significance of anemia in patients with hypothyroidism. Worldwide, 20%-60% of this population may develop anemia, depending on disease severity and nutritional status [7]. A high prevalence of anemia, predominantly normocytic and microcytic, has been reported in patients with hypothyroidism in Pakistan and Saudi Arabia [8-10]. Additionally, a 2024 study observed an association between anemia severity and thyroid hormone levels, underscoring the need for regional studies [8]. Macrocytic anemia is less common but remains clinically relevant because of its association with autoimmune comorbidities and vitamin B12 deficiency [4].

Anemia in patients with hypothyroidism is largely attributed to the physiological effects of reduced thyroid hormone levels, which impair erythropoietin production, slow erythroid proliferation, and alter iron metabolism. Despite this well-established mechanism, the prevalence and hematological patterns of anemia in hypothyroidism vary across populations, and only limited data are available from the Qassim region. Therefore, the primary objective of this study was to determine the prevalence of anemia in patients with hypothyroidism in the region. The secondary objectives were to characterize the hematological patterns of anemia, evaluate the association between anemia and thyroid hormone status, and identify independent predictors of anemia using multivariable logistic regression.

## Materials and methods

Study design and setting

This retrospective observational study used a convenience sampling technique and included patients attending the outpatient clinic at King Fahad Specialist Hospital with a diagnosis of hypothyroidism from January 2024 to December 2025.

Inclusion and exclusion criteria

Patients included in the study had primary hypothyroidism diagnosed according to the diagnostic criteria of the American Thyroid Association and American Association of Clinical Endocrinologists. Primary hypothyroidism was defined as elevated TSH levels above the laboratory reference range (>4.0 mIU/L), low free T4 levels (<9 nmol/L), and clinical signs or symptoms of hypothyroidism [11].

Patients were excluded if they had secondary hypothyroidism; chronic diseases known to cause anemia (e.g., chronic kidney disease, active malignancy, hematological diseases); conditions with bleeding such as peptic ulcers, hemorrhoids, menorrhagia, or abnormal uterine bleeding; pregnancy; antithyroid medication use; or incomplete hospital records.

Sample size

A total of 444 patients with hypothyroidism were included in the final analysis after applying inclusion and exclusion criteria. Ten patients were excluded: three were pregnant, three had chronic kidney disease, and four had incomplete complete blood count results.

Data collection and variables

Data were extracted from patient records and recorded in an Excel spreadsheet (Microsoft Corp., Redmond, WA, USA). Collected variables included demographics (age and sex), chronic diseases (diabetes mellitus, hypertension, ischemic heart disease, heart failure, chronic obstructive pulmonary disease, asthma, and chronic liver disease), smoking status, and history of thyroid cancer. Laboratory data included complete blood count and TSH, T3, and T4 levels. Laboratory measurements were obtained from the most recent outpatient visit at which all required laboratory investigations were available.

Thyroid status was classified as hypothyroid (TSH >4.0 mIU/L), euthyroid (0.4-4.0 mIU/L), or hyperthyroid (TSH <0.4 mIU/L). According to World Health Organization guidelines, anemia was defined as hemoglobin <13.0 g/dL in males and <12.0 g/dL in females. Based on mean corpuscular volume (MCV), anemia was classified as normocytic (80-100 fL), microcytic hypochromic (<80 fL), or macrocytic (>100 fL) [12,13].

Confounding variables

Confounders included age, sex, diabetes mellitus, and hypertension, as these factors may independently influence anemia status. These variables were adjusted for in multivariate regression analysis.

Ethical approval

Approval was obtained from the hospital administration and the Regional Research Ethics Committee of Qassim Province (approval number: H-04-Q-001).

Statistical analysis

All statistical analyses were performed using SPSS Statistics version 26 (IBM Corp. Released 2019. IBM SPSS Statistics for Windows, Version 26.0. Armonk, NY: IBM Corp.). Descriptive statistics were presented as frequencies and percentages (N, %) for categorical variables and as mean ± standard deviation (SD) for continuous variables. Associations between anemia status and categorical variables, including age, sex, diabetes, hypertension, thyroid status, and other comorbidities, were assessed using the chi-square test. Based on these significant associations, multivariate regression analysis was performed, adjusting for age, sex, diabetes, and hypertension, to identify the independent predictors of anemia. Adjusted odds ratios (AORs) and corresponding 95% confidence intervals (CIs) were also reported. Statistical significance was set at p < 0.05 for all analyses.

## Results

We analyzed 444 patients diagnosed with hypothyroidism. Table 1 summarizes the demographic and clinical characteristics of the study population by anemia status. The cohort was predominantly female (389; 87.6%). The largest age group comprised patients aged over 50 years (143; 32.2%), followed by those aged 41-50 years (113; 25.5%). Additionally, 117 patients (26.4%) had chronic diseases. The most common chronic condition was diabetes (68; 15.3%), followed by hypertension (51; 11.5%) and a history of thyroid cancer (27; 6.1%). Regarding thyroid status, the majority were euthyroid (229; 51.6%), followed by hypothyroid (171; 38.5%) and hyperthyroid (44; 9.9%). When comparing anemic (112; 25.2%) and non‑anemic (332; 74.8%) patients, most demographic variables showed no significant differences. Age distribution was similar across the groups (p = 0.664), and neither diabetes (p = 0.118) nor hypertension (p = 0.283) differed significantly. However, sex distribution showed a significant association with anemia, with females comprising a higher proportion of anemic patients (106; 94.6%) compared with non‑anemic patients (283; 85.2%) (χ² = 6.821, p = 0.009). Asthma was more common in anemic patients (5; 4.5%) than in non-anemic patients (2; 0.6%) (p = 0.005). However, this association may be influenced by the small number of asthmatic patients in the cohort (7; 1.6%). Additionally, the presence of any associated chronic disease was significantly higher in the anemic group (40; 35.7%) compared with the non‑anemic group (77; 23.2%) (χ² = 6.766, p = 0.009). Hypothyroidism tended to be more common in anemic patients (52; 46.4%) than non-anemic patients (119; 35.8%), although this was not statistically significant (p = 0.093). Other comorbidities, including ischemic heart disease, heart failure, smoking, and a history of thyroid cancer, were similar between the groups (all p > 0.05).

**Table 1:** Demographic and clinical characteristics of patients in relation to anemia Results are presented as frequencies (n) and percentages (%) or as mean ± SD. § p-values were calculated using the chi-square test. For cells with an expected frequency of <5, the chi-square statistic (χ²) was not calculated; therefore, an exact test was used to determine the p-value. ** Statistically significant at p < 0.05. NA: not applicable, SD: standard deviation

Study variables	Overall N (%) (n = 444)	Patient group		χ2	p-value ^§^
		Anemic N (%) (n = 112)	Non-anemic N (%) (n = 332)		
Age group					
≤30 years	85 (19.1%)	18 (16.1%)	67 (20.2%)	1.582	0.664
31-40 years	103 (23.2%)	30 (26.8%)	73 (22.0%)		
41-50 years	113 (25.5%)	28 (25.0%)	85 (25.6%)		
>50 years	143 (32.2%)	36 (32.1%)	107 (32.2%)		
Sex					
Male	55 (12.4%)	6 (5.4%)	49 (14.8%)	6.821	0.009 **
Female	389 (87.6%)	106 (94.6%)	283 (85.2%)		
Diabetes					
No	376 (84.7%)	100 (89.3%)	276 (83.1%)	2.445	0.118
Yes	68 (15.3%)	12 (10.7%)	56 (16.9%)		
Hypertension					
No	393 (88.5%)	96 (85.7%)	297 (89.5%)	1.154	0.283
Yes	51 (11.5%)	16 (14.3%)	35 (10.5%)		
Ischemic heart disease					
No	436 (98.2%)	108 (96.4%)	328 (98.8%)	NA	0.115
Yes	8 (1.8%)	4 (3.6%)	4 (1.2%)		
Heart failure					
No	442 (99.5%)	111 (99.1%)	331 (99.7%)	NA	0.441
Yes	2 (0.5%)	1 (0.9%)	1 (0.3%)		
Asthma					
No	437 (98.4%)	107 (95.5%)	330 (99.4%)	NA	0.005 **
Yes	7 (1.6%)	5 (4.5%)	2 (0.6%)		
History of thyroid cancer					
No	417 (93.9%)	103 (92.0%)	314 (94.6%)	1.002	0.317
Yes	27 (6.1%)	9 (8.0%)	18 (5.4%)		
Smoking					
No	440 (99.1%)	112 (100%)	328 (98.8%)	NA	0.576
Yes	4 (0.9%)	0	4 (1.2%)		
Associated chronic disease					
No	327 (73.6%)	72 (64.3%)	255 (76.8%)	6.766	0.009 **
Yes	117 (26.4%)	40 (35.7%)	77 (23.2%)		
Thyroid status					
Euthyroid	229 (51.6%)	48 (42.9%)	181 (54.5%)	4.742	0.093
Hyperthyroid	44 (9.9%)	12 (10.7%)	32 (9.6%)		
Hypothyroid	171 (38.5%)	52 (46.4%)	119 (35.8%)		

**Figure 1:**
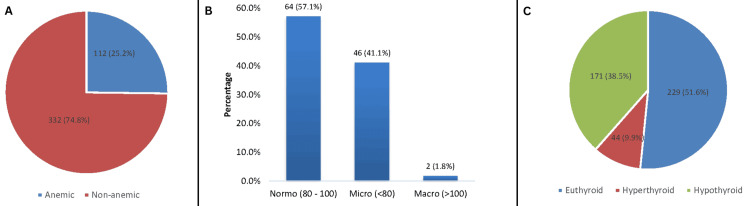
(A) Prevalence of anemia, (B) MCV classification, and (C) distribution of thyroid status in patients with hypothyroidism MCV: mean corpuscular volume

Figure 1 shows that the prevalence of anemia in this cohort was 25.2% (112 patients), while the remaining 332 patients (74.8%) were non-anemic. In anemic patients (112; 25.2%), the majority had normocytic MCV values (64; 57.1%), followed by a substantial proportion with microcytosis (46; 41.1%), whereas macrocytosis was uncommon (2; 1.8%). The thyroid status distribution showed that 229 (51.6%) of the patients were euthyroid, 171 (38.5%) were hypothyroid, and 44 (9.9%) were hyperthyroid.

The metabolic and laboratory characteristics of the study population stratified by anemia status are shown in Table 2. Mean values for most hematological and thyroid parameters fell within expected physiological ranges, including WBC count (7.34 ± 2.28 × 10⁹/L), RBC count (4.59 ± 0.53 × 10¹²/L), hemoglobin (12.8 ± 1.53 g/dL), MCV (86.2 ± 7.46 fL), mean corpuscular hemoglobin (MCH) (27.8 ± 2.99 pg), mean corpuscular hemoglobin concentration (MCHC) (32.1 ± 1.53 g/dL), hematocrit (39.4 ± 4.61%), red cell distribution width (RDW) (13.8 ± 1.61%), and platelet count (309.9 ± 87.1 × 10⁹/L). Thyroid function markers showed wide variability, with a mean TSH of 5.28 ± 9.52 µIU/mL, T3 of 13.7 ± 2.91 nmol/L, and T4 of 4.07 ± 0.74 nmol/L. When comparing anemic (112; 25.2%) and non‑anemic (332; 74.8%) patients, several hematological indices showed highly significant differences. As expected, anemic patients had markedly lower RBC count (4.22 ± 0.49 vs. 4.72 ± 0.48 × 10¹²/L; p < 0.001), hemoglobin (10.9 ± 0.91 vs. 13.4 ± 1.07 g/dL; p < 0.001), hematocrit (34.1 ± 2.94 vs. 41.2 ± 3.58%; p < 0.001), MCV (81.5 ± 8.74 vs. 87.8 ± 6.21 fL; p < 0.001), MCH (25.9 ± 3.36 vs. 28.5 ± 2.55 pg; p < 0.001), MCHC (31.6 ± 1.92 vs. 32.3 ± 1.33 g/dL; p < 0.001), and RDW (15.1 ± 1.99% vs. 13.4 ± 1.19%; p < 0.001). Platelet count was significantly higher in anemic patients (336.3 ± 100 vs. 300.9 ± 80.3 × 10⁹/L; p < 0.001). In contrast, the WBC count (p = 0.111) and thyroid function markers (TSH, p = 0.233; T3, p = 0.99; T4, p = 0.097) did not differ significantly between the groups.

**Table 2:** Metabolic and laboratory characteristics in relation to anemia prevalence Results are presented as mean ± SD. § p-values were calculated using the independent-samples t-test. ** Statistically significant at p < 0.05. WBC: white blood cell count, RBC: red blood cell count, Hb: hemoglobin, MCV: mean corpuscular volume, MCH: mean corpuscular hemoglobin, MCHC: mean corpuscular hemoglobin concentration, HCT: hematocrit, RDW: red cell distribution width, PLT: platelet count, TSH: thyroid-stimulating hormone, T3: triiodothyronine, T4: thyroxine, SD: standard deviation

Variables	Overall mean ± SD (n = 444)	Patient group		T-test	p-value ^§^
		Anemic mean ± SD (n = 112)	Non-anemic mean ± SD (n = 332)		
WBC (×10⁹/L)	7.34 ± 2.28	7.04 ± 2.17	7.44 ± 2.31	1.599	0.111
RBC count (×10¹²/L)	4.59 ± 0.53	4.22 ± 0.49	4.72 ± 0.48	9.228	<0.001 **
Hb (g/dL)	12.8 ± 1.53	10.9 ± 0.91	13.4 ± 1.07	23.049	<0.001 **
MCV (fL)	86.2 ± 7.46	81.5 ± 8.74	87.8 ± 6.21	8.442	<0.001 **
MCH (pg)	27.8 ± 2.99	25.9 ± 3.36	28.5 ± 2.55	8.527	<0.001 **
MCHC (g/dL)	32.1 ± 1.53	31.6 ± 1.92	32.3 ± 1.33	4.191	<0.001 **
HCT (%)	39.4 ± 4.61	34.1 ± 2.94	41.2 ± 3.58	18.888	<0.001 **
RDW (%)	13.8 ± 1.61	15.1 ± 1.99	13.4 ± 1.19	10.607	<0.001 **
PLT (×10⁹/L)	309.9 ± 87.1	336.3 ± 100	300.9 ± 80.3	3.763	<0.001 **
TSH (µIU/mL)	5.28 ± 9.52	6.23 ± 10.9	4.97 ± 9.01	1.194	0.233
T3 (nmol/L)	13.7 ± 2.91	13.3 ± 2.97	13.8 ± 2.88	1.655	0.099
T4 (nmol/L)	4.07 ± 0.74	3.92 ± 0.92	4.12 ± 0.67	1.666	0.097

Table 3 presents the results of the multivariate logistic regression analysis of the independent variables for anemia in patients with hypothyroidism. Some variables were substantially associated with an increased risk of anemia after controlling for age, sex, diabetes, and hypertension. Female sex was a significant predictor; the odds of anemia in females were more than three times that in males (AOR = 3.210, 95% CI: 1.323-7.789, p = 0.010). Additionally, the odds of anemia were more than three times higher in patients with concomitant chronic diseases (AOR = 3.458, 95% CI: 1.829-6.536, p < 0.001). These findings indicate that sex and concomitant chronic diseases are independently associated with the risk of anemia in this hypothyroid population.

**Table 3:** Multivariate regression analysis to determine the predictors of anemia in patients with hypothyroidism (n = 444) Adjusted for age, sex, diabetes, and hypertension. ** Statistically significant at p < 0.05. AOR: adjusted odds ratio, CI: confidence interval

Factor	AOR	95% CI	p-value
Sex			
Male	Ref		
Female	3.21	1.323-7.789	0.010 **
Associated chronic disease			
No	Ref		
Yes	3.458	1.829-6.536	<0.001 **

## Discussion

In the 444 patients with hypothyroidism included in the study, 112 (25.2%) had anemia, confirming the recognized hematological consequences of thyroid dysfunction. This demographic profile of predominantly female and middle-aged patients is consistent with the global epidemiological pattern of higher prevalence of hypothyroidism in women and older adults [1,2]. There were no significant differences in most demographic variables between the anemic and non‑anemic groups; however, female sex was independently associated with anemia. This is consistent with previous studies showing that women with hypothyroidism are more likely to develop anemia because of menstrual blood loss, a higher prevalence of autoimmune thyroiditis, and nutritional deficiencies [3,4].

The prevalence of anemia in our cohort (25.2%) is comparable to the 20-30% reported in previous studies [3,7,8], but lower than the frequencies reported in South Asian and African populations, where the prevalence of anemia in patients with hypothyroidism is between 40% and over 60% [4,9,14-17]. Anand et al. reported a prevalence of 41% in India [14], whereas Bhattacharya et al. reported anemia in 52% of patients with hypothyroidism in Eastern India [15]. Anemia rates above 45% have also been reported by Bilonia et al. and Bobbili et al. [16,17]. These differences may reflect regional variations in dietary iron intake, vitamin B12 deficiency, socioeconomic status, and iodine adequacy. For example, vitamin B12 deficiency is common in South Asian populations owing to their dietary habits and has been associated with a higher prevalence of anemia [4,15]. Furthermore, deficiencies in micronutrients such as iron and selenium are known to affect thyroid hormone synthesis and erythropoiesis, thereby aggravating anemia in resource-limited settings [6].

In our cohort, normocytic anemia was the most common type (57.1%), followed by microcytic anemia (41.1%), whereas macrocytosis was rare. This pattern is consistent with the classical description of anemia associated with hypothyroidism, which is typically normocytic due to decreased erythropoietin production and impaired erythroid maturation [5]. The predominant pattern was normocytic anemia, similar to that reported by Mehmet et al. [3], Das and Chakravarty [18], and Patel and Jain [19]. However, microcytic anemia was found to be the most common type in some studies, particularly in areas with a high prevalence of iron deficiency [8,10,16,20]. Gul et al. showed that the prevalence of microcytosis was higher in subclinical hypothyroidism than in euthyroidism [20]. Mandefro et al. reported microcytic anemia as the predominant pattern in Ethiopia, attributed to widespread nutritional deficiencies [21]. These differences highlight the influence of regional nutritional status, chronic disease burden, and underlying etiologies in the morphology of anemia.

The higher platelet counts observed in our anemic patients were consistent with reactive thrombocytosis, a common finding in iron-deficiency anemia. This finding is consistent with the pathophysiological interactions between iron metabolism and hematopoiesis described in previous studies [6]. There were no significant differences in the anemic and non-anemic groups in thyroid function parameters (TSH, T3, and T4). This suggests that anemia can occur across different thyroid hormone levels and that anemia may take time to develop or resolve in response to these hormones. This is consistent with studies by Floriani et al. [7] and Shah et al. [22], who reported that anemia may be observed in patients with mild or subclinical thyroid dysfunction.

The association between chronic diseases and anemia is well recognized, as systemic inflammation and comorbidities can suppress erythropoietin release and/or impair iron utilization [11]. These findings are consistent with those of Mandefro et al. [21], who found that chronic illness is an important determinant of anemia in thyroid disorders.

Our findings are generally consistent with the global literature but also reveal region-specific patterns, particularly the predominance of normocytic anemia and the influence of chronic disease. Variation between studies may be related to population characteristics, iodine status, nutritional patterns, prevalence of autoimmune thyroiditis, and methodological differences in defining anemia and thyroid dysfunction. The independent predictors of female sex and chronic diseases highlight the need for targeted screening and early intervention in high-risk subgroups. Additionally, the global burden of nutritional anemia remains significant. Recent hospitalization data from the United States indicate that the morbidity associated with anemia remains substantial [23], highlighting the importance of integrated management strategies for patients with thyroid dysfunction.

Study strengths

This study has several important strengths. First, it includes a relatively large sample size, which enhances the statistical power and reliability of the findings. Second, anemia was defined using standardized World Health Organization criteria, ensuring consistency and comparability with other studies. Third, the use of multivariable logistic regression analysis allowed adjustment for key confounding factors, improving the validity of the observed associations. Finally, this study contributes valuable regional data from Saudi Arabia, addressing an important gap in the literature regarding the hematological profile of patients with hypothyroidism in this population.

Study limitations

This study has several limitations that should be considered when interpreting the findings. First, its retrospective design prevented the establishment of causal associations between hypothyroidism, comorbidities, and anemia. Second, because this was a single-center study, the results may not be generalizable to populations with different demographic, nutritional, and socioeconomic characteristics. Third, we could not determine the specific causes of anemia due to the lack of key biochemical markers, including ferritin, transferrin saturation, vitamin B12, and folate. Furthermore, although patients with major known causes of anemia, such as chronic kidney disease, active bleeding, and hematological disorders, were excluded, residual confounding from other comorbidities may still have influenced the study findings.

Additionally, although multivariable logistic regression was used to adjust for key confounders, residual and unmeasured confounding cannot be excluded given the retrospective nature of the study and the limited availability of variables. The assessment of thyroid hormone levels at a single time point may not adequately reflect the duration or chronicity of hypothyroidism, which could limit the interpretation of the association between thyroid hormone levels and anemia. Finally, the absence of longitudinal follow-up precludes the assessment of the anemia response to thyroid hormone replacement therapy or the correction of nutritional deficiencies.

## Conclusions

The present study shows that anemia is a common comorbidity in patients with hypothyroidism and affects one in four patients. The predominant pattern was normocytic anemia; however, microcytosis was also frequent. The presence of chronic diseases and female sex were significant independent predictors of anemia, emphasizing the multifactorial nature of hematological derangements associated with thyroid dysfunction. There was no significant difference in thyroid hormone levels between the groups; however, this finding should be interpreted cautiously, as thyroid hormone levels were assessed at a single time point and may not reflect disease chronicity. However, the strong association with comorbid conditions suggests the need for a thorough clinical evaluation. These findings emphasize the need for tailored treatment strategies in patients with hypothyroidism to improve clinical outcomes and reduce disease burden.
